# Remote Data Collection During a Pandemic: A New Approach for Assessing and Coding Multisensory Attention Skills in Infants and Young Children

**DOI:** 10.3389/fpsyg.2021.731618

**Published:** 2022-01-21

**Authors:** Bret Eschman, James Torrence Todd, Amin Sarafraz, Elizabeth V. Edgar, Victoria Petrulla, Myriah McNew, William Gomez, Lorraine E. Bahrick

**Affiliations:** ^1^Department of Psychology, Florida International University, Miami, FL, United States; ^2^Center for Children and Families, Florida International University, Miami, FL, United States; ^3^University of Miami Institute for Data Science and Computing, Miami, FL, United States

**Keywords:** gaze estimation, online data collection, remote data collection, looking time, Gorilla Experiment Builder, OpenFace, machine learning

## Abstract

In early 2020, in-person data collection dramatically slowed or was completely halted across the world as many labs were forced to close due to the COVID-19 pandemic. Developmental researchers who assess looking time (especially those who rely heavily on in-lab eye-tracking or live coding techniques) were forced to re-think their methods of data collection. While a variety of remote or online platforms are available for gathering behavioral data outside of the typical lab setting, few are specifically designed for collecting and processing looking time data in infants and young children. To address these challenges, our lab developed several novel approaches for continuing data collection and coding for a remotely administered audiovisual looking time protocol. *First*, we detail a comprehensive approach for successfully administering the Multisensory Attention Assessment Protocol (MAAP), developed by our lab to assess multisensory attention skills (MASks; duration of looking, speed of shifting/disengaging, accuracy of audiovisual matching). The MAAP is administered from a distance (remotely) by using Zoom, Gorilla Experiment Builder, an internet connection, and a home computer. This new data collection approach has the advantage that participants can be tested in their homes. We discuss challenges and successes in implementing our approach for remote testing and data collection during an ongoing longitudinal project. *Second*, we detail an approach for estimating gaze direction and duration collected remotely from webcam recordings using a post processing toolkit (OpenFace) and demonstrate its effectiveness and precision. However, because OpenFace derives gaze estimates without translating them to an external frame of reference (i.e., the participant's screen), we developed a machine learning (ML) approach to overcome this limitation. Thus, *third*, we trained a ML algorithm [(artificial neural network (ANN)] to classify gaze estimates from OpenFace with respect to areas of interest (AOI) on the participant's screen (i.e., left, right, and center). We then demonstrate reliability between this approach and traditional coding approaches (e.g., coding gaze live). The combination of OpenFace and ML will provide a method to automate the coding of looking time for data collected remotely. Finally, we outline a series of best practices for developmental researchers conducting remote data collection for looking time studies.

## Introduction

In early 2020, in-person participant testing and data collection dramatically slowed or was completely halted across the world as some labs were forced to close due to the COVID-19 pandemic. Developmental researchers who assess looking time (especially those who rely heavily on in-lab eye-tracking or live observer coding) were forced to re-think their methods of data collection. They could either analyze old data *or* they could attempt to adapt their data collection techniques to remote testing platforms—e.g., online data collection using an internet-connected computer in the child's home. During March of 2020, our lab was forced to close its doors to in-person participant testing in the middle of an extended longitudinal project. In an effort to continue data collection, we adapted many of our “in-lab” protocols and tasks to a format suitable for a remote setting. We found it relatively easy to convert parent questionnaires and assessments of children's language, social, and cognitive functioning, to this format. However, collecting looking time data for audiovisual tasks (i.e., tasks that track infant attention to multiple dynamic visual events in the presence of a soundtrack matching one of them) including the Multisensory Attention Assessment Protocol (MAAP; Bahrick et al., 2018a), posed significant challenges. For example, there are large individual differences in participants' home computer setups (e.g., differences in screen size, web camera quality, lighting, internet speed, etc.), making it difficult to use webcam-based eye-tracking techniques or to reliably code gaze in real-time. Further, because offline coding from videos (e.g., frame-by-frame) is time- and labor-intensive, we wanted to find a solution that might expedite the data coding process.

Fortunately, for those like us who opted to continue data collection during the pandemic, there are a variety of remote or online platforms (e.g., Amazon Mechanical Turk, Gorilla, Lookit, PyHab, etc.) that are specifically designed for gathering behavioral data outside of the typical lab setting. For example, Lookit has shown significant promise for remote data collection of looking time from infants and children (e.g., Scott and Schulz, 2017). It provides a secure, robust platform that can translate developmental methods to a computer-based home testing environment, affording greater accessibility to families both within and outside the university community. Similarly, Gorilla Experiment Builder is a promising tool for online data collection in adults and children, particularly for assessing executive functioning and working memory (Anwyl-Irvine et al., 2018; Ross-Sheehy et al., 2021), and it can also be used for collecting data from looking time tasks. While these platforms provide developmental researchers with legitimate options for online data collection, they have yet to be thoroughly vetted and tested with infants and children in the home, integrated with audiovisual tasks, or integrated with reliable methods for gaze coding for audiovisual tasks.

For our purposes, we opted to use Gorilla Experiment Builder for the following reasons: (1) it provides excellent control of temporal parameters (e.g., trial onsets and offsets), (2) optimal video playback (e.g., little lagging, synchronous audio, and video playback), (3) an intuitive interface for building experiments so that they can be quickly deployed for remote data collection, and (4) can easily and efficiently be deployed in the home with minimal technology (e.g., experiments can be accessed through a web browser; Anwyl-Irvine et al., 2020a,b). In this paper, we detail our approach for collecting and coding looking time data remotely from our MAAP protocol—a three-screen (left, right, center displays) individual difference measure of three foundational attention skills (duration of looking, speed of shifting/disengaging, accuracy of audiovisual matching) to audiovisual social and non-social events (Bahrick et al., 2018a)—using widely available software and hardware available on home computers. We then describe our approach for scoring data from the MAAP that have been collected in the home, using a newly developed platform for estimating gaze behavior from video recordings (OpenFace), as well as our development of a machine learning (ML) model to translate the estimates provided by OpenFace into meaningful looking time data (i.e., left, right, and center displays). We end by discussing the implications this new approach for developmental researchers who are interested in collecting looking time data from infants and children remotely.

### Traditional Methods for Coding Looking Time for Infants and Children

Developmental researchers who use looking time as an index of infant perception or cognition typically code it in one of three ways: using frame-by-frame coding, coding gaze in real time, or by using one of many different types of eye trackers. The general goal in using all of these methods is to estimate where the participant is looking on a screen, when they initiated the look (look onset), and how long they remain fixated on a particular location (look duration and offset). Typically, researchers define multiple areas of interest (AOIs) to demarcate locations on a screen displaying visual images that participants could view. For example, researchers have assessed looking time to the entire screen (e.g., Richards, 1987; Lewkowicz, 1988; Colombo et al., 1991), or locations corresponding to multiple images/events on a single screen (e.g., Hirsh-Pasek and Golinkoff, 1996; Bahrick et al., 2018b). While together, these looking time methods have generated a tremendous amount of information about the development of attention, perception, and cognition, they require training coders (e.g., live coding and frame-by-frame coding), can be time consuming (e.g., frame-by-frame coding), and in some instances, cannot be adapted to an online setting (e.g., remote eye-tracking). The following is a brief overview of each of these methods for coding looking time and the problems that might arise when applied to coding data collected online.

#### Frame-by-Frame Coding

Frame-by-frame coding involves estimating gaze direction on each frame from a video recording (Fernald et al., 2008; Ross-Sheehy et al., 2015). Estimates of inter-rater reliability between human observers is typically very high (e.g., 85–95% agreement) but there appear to be limitations to the number of locations that can be reliably coded. This is potentially due to the relatively low spatial and temporal resolution when coding gaze direction from videos (Wass et al., 2013), making it difficult to code looking to more than two or three locations. These limitations are especially evident for data collected remotely as several of the environmental constraints that the lab setting affords (e.g., standardization of distance, position, and lighting) are absent. Further, this method of coding is extremely time consuming, and human coders can take up to 5 h to code 10 min of video (Wass et al., 2013). Due to this, frame-by-frame coding limits the amount of data that can be processed, and is typically used for shorter tasks (e.g., Jesse and Johnson, 2016). Further, human observers need to be trained and reliability must be established, both of which are also time consuming (Oakes, 2012).

#### Live Coding

Coding gaze in real time by trained observers is a widespread method for quantifying looking time. Observers, blind to the conditions of the study and unable to see the presentation of visual stimuli, estimate gaze direction and duration in real time while the participant views the stimuli (e.g., Lewkowicz, 1988; Bahrick et al., 2018a). This method is more time-efficient than frame-by-frame coding and requires little post processing of the data. In addition, if needed, observers can also code gaze offline from a video recording of the data collection. This approach has been used to estimate looking to a single location on a screen (e.g., Bahrick and Lickliter, 2000; Shaddy and Colombo, 2004; Altvater-Mackensen et al., 2016), looking to two locations (e.g., left, right; Bahrick and Watson, 1985; Bahrick, 1987; Casey and Richards, 1988), or looking to three locations (e.g., left, center, right; Bahrick et al., 2018a). However, for assessments administered remotely, coding data in real time is prohibitively difficult and offline coding of a video recorded via a webcam from a home computer can also be challenging. Specifically, without a clear frame of reference, it is difficult to judge where on the screen the participant is looking and how well the participant's looking is time-locked with the onsets and offsets of the visual stimulus events. Further, procedures should be used to ensure that observers are unaware of the experimental conditions (Oakes, 2012), which can also be difficult using online platforms.

#### Eye-Tracking

Eye-tracking has become an increasingly popular tool for examining looking time and has been developed and refined over the last several decades (e.g., Hutton, 2019). Compared to methods using human observers, eye-tracking allows researchers to obtain gaze location objectively without the need to manually code the data (Hessels and Hooge, 2019) and features higher temporal and spatial resolution for gathering samples (Aslin, 2012; Wass et al., 2013). Gaze direction is determined by the reflection of infrared light sources on the eye(s) using information from the calibration process conducted prior to data collection. The calibration process stores information about the participant's pupil(s) and corneal reflection(s) for fixations at specified locations on the screen (Oakes, 2010, 2012). This allows for gaze to be measured in terms of X, Y coordinates for any location on the screen. However, infrared eye-tracking cannot be employed for remote data collection. Though webcam-based eye-trackers show some promise for remote data collection (Semmelmann and Weigelt, 2017), there has been little research into the feasibility of their use in collecting gaze data from infants and young children. For example, changes in participant's head position can lead to significant data loss, and calibration can be tedious and time consuming (increasing the likelihood of participant fatigue).

In sum, while the techniques reviewed above have provided a wealth of information derived from the looking behavior of infants and young children, they were not optimal (and in some instances, not possible) for our purposes of coding looking time from a protocol administered remotely. We sought to devise an approach in which looking time to a multi-screen audiovisual protocol (the MAAP; see section Our Audiovisual Task: The Multisensory Attention Assessment Protocol) could be coded accurately and efficiently across many participants. Thus, our approach does not supplant prior approaches, but rather provides researchers with a new tool for coding looking time data, one that is optimized for remote data collection. We detail our new approach in sections Objective 1: Data Collection at a Distance, Objective 2: Using OpenFace to Derive Gaze Estimates From Web-Cam Recordings, and Objective 3: Training a ML Model to Calculate Looking Time Data.

### Online Data Collection

In addition to the challenges of coding looking time in an online setting, there are a number of challenges specific to online data collection. For example, a unique problem with online (internet-based) testing is its reliance on participants' home computer hardware and software. In the lab, researchers develop and refine their lab computer, stimulus software, and hardware for data collection. More important, they can be sure that all participants are tested using the same system. For online testing, the opposite is true: participants use different computers (desktop, laptop, tablet, or even phone), as well as different operating systems and web browsers. Because of this, ensuring uniform standards for data collection is extremely difficult. While all of the unique combinations of hardware and software are not equal, some home computer set-ups outperform others (Anwyl-Irvine et al., 2020a,b). By limiting the number of platforms (described below), designing experiments that require minimum amounts of technology, and providing the participants/caregivers with explicit detail on how to set up their home computer, we can more closely recreate the lab setting using remote testing in the home.

### Post-processing of Looking Time Data

Recent advances in the offline post processing of looking time data provide researchers with a viable option for scoring data collected remotely (as well as in the lab) from video recordings. In fact, there are a wide variety of open-source tools and commercial systems available for eye-gaze estimation (Wood and Bulling, 2014; Baltrusaitis et al., 2016; Park et al., 2018; Chouinard et al., 2019) and webcam-based eye tracking solutions (e.g., https://github.com/stepacool/Eye-Tracker and https://webgazer.cs.brown.edu/). Further, Chouinard et al. (2019) used an automatic face analysis tool (Amazon Rekognition) to automate infant preferential looking coding from video data collected online.

**Figure 1:**
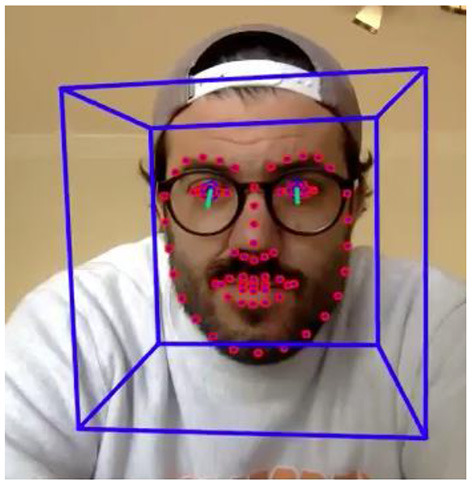
Example of OpenFace output including facial landmark detection, head pose estimation, facial action unit recognition, and eye-gaze estimation. These metrics are computed for each frame of the video. Depicted is a single frame from the video.

We found that one tool in particular (OpenFace; https://github.com/TadasBaltrusaitis/OpenFace#eye-gaze-tracking) seems to be well-suited to address our specific needs of coding looking time data collected in a remote setting (for our particular three-screen audio visual task) with infants and young children. We chose to use OpenFace for eye-gaze estimation in our current project for the following reasons. OpenFace is an open source, post processing, gaze estimation tool (the code is freely available for academic purposes). It is the first toolkit with available source code capable of facial landmark detection, head pose estimation, facial action unit recognition, and, most importantly, eye-gaze estimation (see Figure 1). Further, this tool can estimate these parameters from video recordings of a participant's face.

Although there has been an increased interest in automatic gaze estimation analysis and understanding, there has been little application of these techniques to infants and young children (but see Chouinard et al., 2019). Further little is known about how these techniques may be integrated with online data collection—using technology owned by most families (e.g., laptops, basic internet, standard webcams).

### The Current Project

The current project has three main objectives. *First*, we lay out a detailed approach for successfully collecting looking time data from a distance. Using only Zoom, an online experiment builder (Gorilla), an internet connection, and equipment typically found in the home (e.g., laptop, webcam, speakers), we have developed a novel, successful method for administering an audiovisual looking time task (the MAAP) remotely. *Second*, we detail an approach for estimating gaze direction and duration collected remotely from webcam recordings using OpenFace. While OpenFace provides us with estimates of gaze direction/vectors, these estimates are meaningless in the absence of an external frame of reference (specific location on the participants screen). *Third*, to overcome this challenge, we developed a novel ML approach for training an algorithm (neural network) to classify gaze direction/vectors into traditional looking time data (e.g., total looking to left, right, and center displays) by relating gaze directions from OpenFace to an external frame of reference (locations on the participant's screen). To assess accuracy of these looking time estimates, we assess reliability between these looking time estimates using OpenFace/ML and the same data that were previously coded live (traditional approach) from a longitudinal study conducted in our lab using the MAAP (Bahrick et al., 2018b). The data that were coded live serve as the baseline and proof of concept for using OpenFace/ML to code looking time data. We then discuss applying this novel approach to data collected in participants' homes from webcam recordings. We provide the ML model with a series of “known locations” (attention getting stimuli), to define looks to left, right, and center. Further, we provide a set of guidelines for how to implement looking time measures in the home, with minimal software and equipment. This novel approach is designed to address the immediate need of continuing data collection during a pandemic (or any lab shutdown) by combining a variety of methods into a single framework. In addition to serving this immediate purpose, it is our hope that this method can be developed further to offer future researchers a viable method for collecting meaningful data remotely.

### Our Audiovisual Task: The Multisensory Attention Assessment Protocol

We demonstrate the effectiveness of this approach to online data collection and the OpenFace post processing method using data collected in our lab from the MAAP (Bahrick et al., 2018a). The MAAP is a fine-grained measure of individual differences in attention to dynamic, audiovisual social, and non-social events, appropriate for infants and young children. The MAAP assesses three multisensory attention skills (MASks; duration of looking, speed of shifting/disengaging, accuracy of audiovisual matching) using 24 short trials (to provide stable means), presents blocks of both social and non-social events, and indexes the cost of competing stimulation from a visual distractor event on each of these skills. Trials of the MAAP consist of a 3-s dynamic, silent central event (morphing geometric shapes) followed by two 12-s side-by-side lateral events of women speaking (social events) or objects impacting a surface in an erratic pattern (non-social events). One of the lateral events is synchronous with its natural soundtrack, and the other lateral event is asynchronous with the soundtrack. For an example video, visit https://nyu.databrary.org/volume/326. Performance on the MAAP predicts language outcomes in typically developing infants and children (Bahrick et al., 2018a; Edgar et al., Under review^1^), and predicts language and symptomatology in children with autism (Todd and Bahrick, Under review)^2^. Also, unlike prior research using static images or silent events, by presenting audiovisual events on three displays, and presenting both social and non-social events in the presence of an irrelevant visual distractor, the MAAP better reflects the natural, multisensory learning environment of the child. Further, the MAAP requires no verbal responses or verbal instructions to the child, and is thus able to provide a common measure for assessing development across infancy and early childhood.

## Objective 1: Data Collection at a Distance

We adapted two well-established remote data collection platforms (Zoom and Gorilla) for use with technology that is commonly found in the home. Zoom provides videoconferencing and online chat services through a cloud-based peer-to-peer software platform (https://zoom.us/). This platform provides a stable environment for real time face-to-face communication, including live interaction allowing the experimenter to provide instructions and guidance, as well as the opportunity for the participants to ask questions or provide feedback. Sessions can be recorded for later behavioral coding. Gorilla (www.gorilla.sc) is an online experiment builder whose aim is to enable researchers to conduct online experiments (regardless of programming and networking knowledge). It provides access to web-based experiments and reduces the risk of introducing noise (e.g., misuse of browser-based technology) in data (Anwyl-Irvine et al., 2018). Combined, these two platforms can be used to conduct looking time tasks in the home with acceptable precision and accuracy for temporal parameters (Anwyl-Irvine et al., 2020a,b).

### Programming and Presenting the Task

After programming a version of the MAAP that ensured sufficient audio-visual synchrony (see Supplementary Material, section 8.1, for details), we used Gorilla Experiment Builder to present it to the participants in their own homes with minimal technical requirements. In addition to providing a platform for programming experiments, Gorilla Experiment Builder provides a straightforward way to present stimuli to participants on their own computer. Gorilla packages the task in a link that can be shared and displayed using a web browser. After the participant clicks on the link, the task will be displayed like a standard web page. Participants are not required to download anything; they simply click, and the program is launched.

### Administering the MAAP Remotely

Although there are methods available to collect looking time data in Gorilla (e.g., webcam based eye-tracking), we found them somewhat difficult to work with and, importantly, the integration of the webcam eye-tracking software with the videos introduced some noise (e.g., lagging videos, asynchrony of video, and audio soundtrack), into the presentation of the MAAP. Because the MAAP depends on ensuring that the audio track aligns with the video, it was imperative that we were able to record looks while maintaining excellent audio-visual precision between the video and the audio track. We found that a combination of Gorilla (not including their webcam-based eye tracking feature) and Zoom provided us with the level of precision that we required. Specifically, our preliminary tests indicated that Gorilla playback through the Zoom share screen function achieved a sufficient level of precision of video and audio playback to allow us to code looking time data from the MAAP. While this sounds simple enough, there are a number of specific settings that the experimenter needed to enable in order to maintain the level of precision needed for this task (see Supplementary Material, section 8.2, for details).

### The Role of the Caregiver

In addition to the technical requirements on the experimenter's end (see Supplementary Material, section 8.3.1), we also found that we needed to ensure that the parent/caregiver had the necessary technology to participate. Because we wanted to maximize time with the child and reduce demands on their attention, we found it helpful to have a “pre-session” with the parent/caregiver to familiarize them with the software (see Supplementary Material, section 8.4, for a description of this pre-session). In this pre-session we assessed their level of comfort with Zoom and familiarized them with its features, if necessary. Next we assessed what kind of computer they were using. While most desktop/laptops were compatible, and Gorilla demonstrates similar performance across both Macs and PCs (Anwyl-Irvine et al., 2020a,b), we found that it was not possible for parents to use tablets or computers that did not have a webcam at the top center of the screen. This is because we needed consistency for our post processing method (e.g., same camera location and distance of the child from the camera). Next, we tested the participant's internet speed. Using https://www.speedtest.net/, we recorded the participant's download speed and their ping rate. We found that as long as their download speed was >50 mbps, and their ping was <25 ms, there were no issues in terms of lagging or asynchronous presentation of the MAAP (see Supplementary Material, section 8.3.2, for more detail).

We also found it imperative to discuss with the parent/caregiver the importance of their role in testing and data collection Specifically, we wanted to emphasize that the parent was not a passive viewer of the data collection, but rather an “active at-home experimenter,” working alongside the experimenters from our lab. By giving parents this title and providing specific instructions for how to best approach the data collection process, we found that parents were more engaged with the data collection process. We started by stressing that they needed to be present for the duration of the session and that they must be ready to help at any time. Helping included: setting up the camera angles, providing technical support (ensuring the tasks opened and were displayed correctly), and keeping the child engaged with the task. We also emphasized that they should not interfere with the data collection and that only the actual experimenter (who was present during task administration to give feedback and instructions) should give feedback to the child. Children were given no instructions about where to look. They were told that they would “watch some videos of ladies talking and objects moving,” that they needed to “sit nice and still,” and that after the video was done, they could “play a *really* fun game.” All of the information in the pre-session was recorded.

## Objective 2: Using OpenFace to Derive Gaze Estimates From Web-Cam Recordings

While the combination of research tools mentioned in section Objective 1: Data Collection at a Distance provides a promising and exciting method for remote data collection, it does not address the issues of quantifying and processing looking time data. Therefore, the video recordings of the participants completing the task require additional post-processing through OpenFace to estimate gaze direction/vectors (for a summary of OpenFace features, see section Post Processing of Looking Time Data). To demonstrate the effectiveness of using OpenFace to estimate gaze direction in infants and young children from a video recording, we first evaluate how well OpenFace can identify the face, head position, and eye gaze direction of the participants. OpenFace provides a confidence rating for every frame of a video (using multiple face, head position, and gaze direction landmarks). The confidence rating is a measure of OpenFace's accuracy in identifying all components. They range from 0 to 100% (higher is better). Frames in which the participant is looking away from the camera or occluding their face would receive a low confidence rating. It should be noted that we did not initially set any criteria for data inclusion. As such, the values provided by OpenFace are raw and unfiltered. Below, we describe the confidence ratings for data collected in the lab (as a proof of concept) and then extended this approach to data that were collected remotely.

### OpenFace Confidence Ratings for Data Collected In-lab

**Figure 2:**
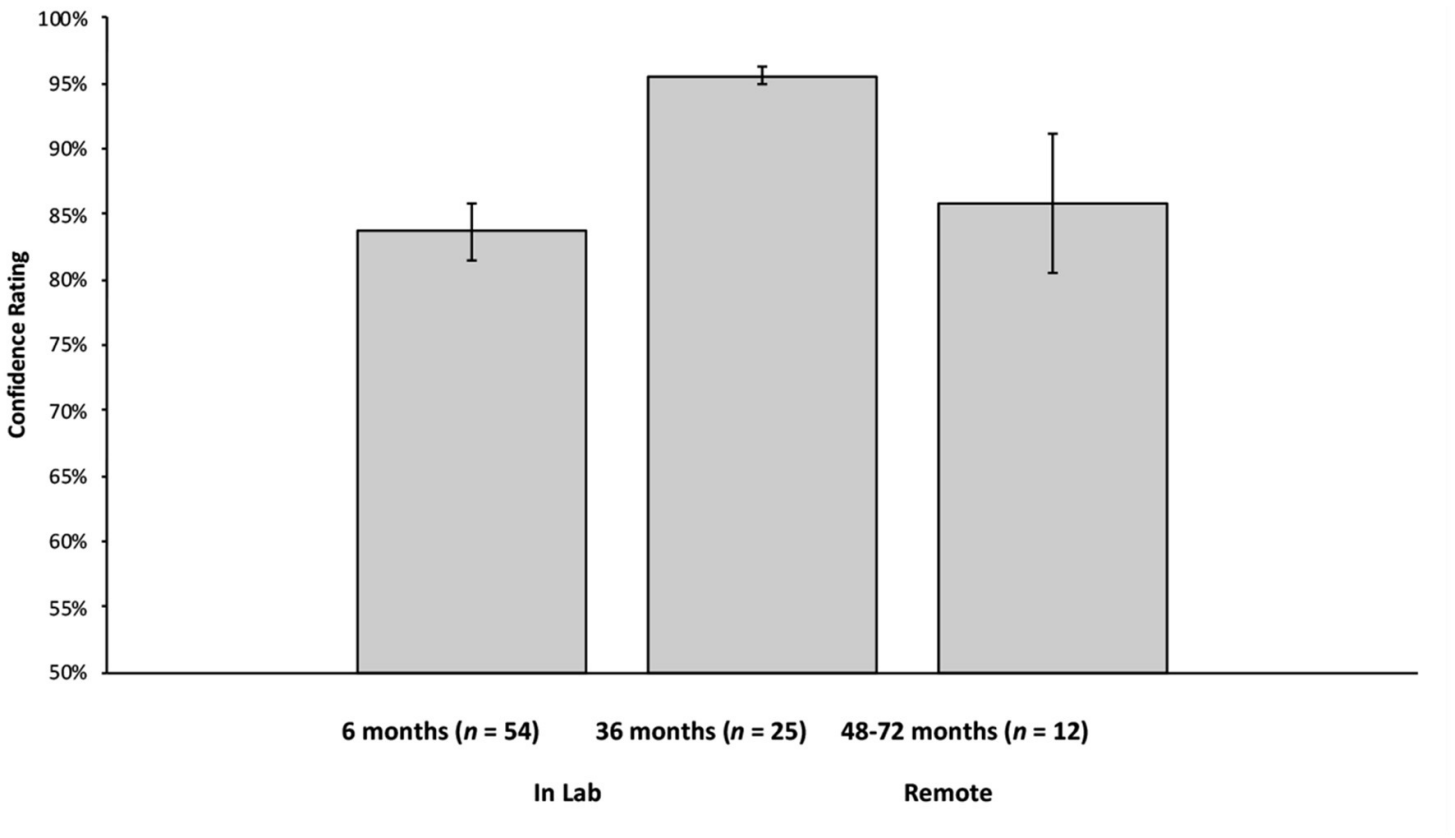
Mean confidence ratings for 6-month-old infants (tested in the lab), 36-month-old children (tested in the lab), and 48–72-month-old children (tested remotely). Error bars reflect standard errors of the means. OpenFace provides a confidence rating for every frame of a video (using all face, head position, and gaze direction landmarks). The confidence rating is a measure of how well OpenFace can identify all of these components (averaged across all frames for each participant).

**Figure 3:**
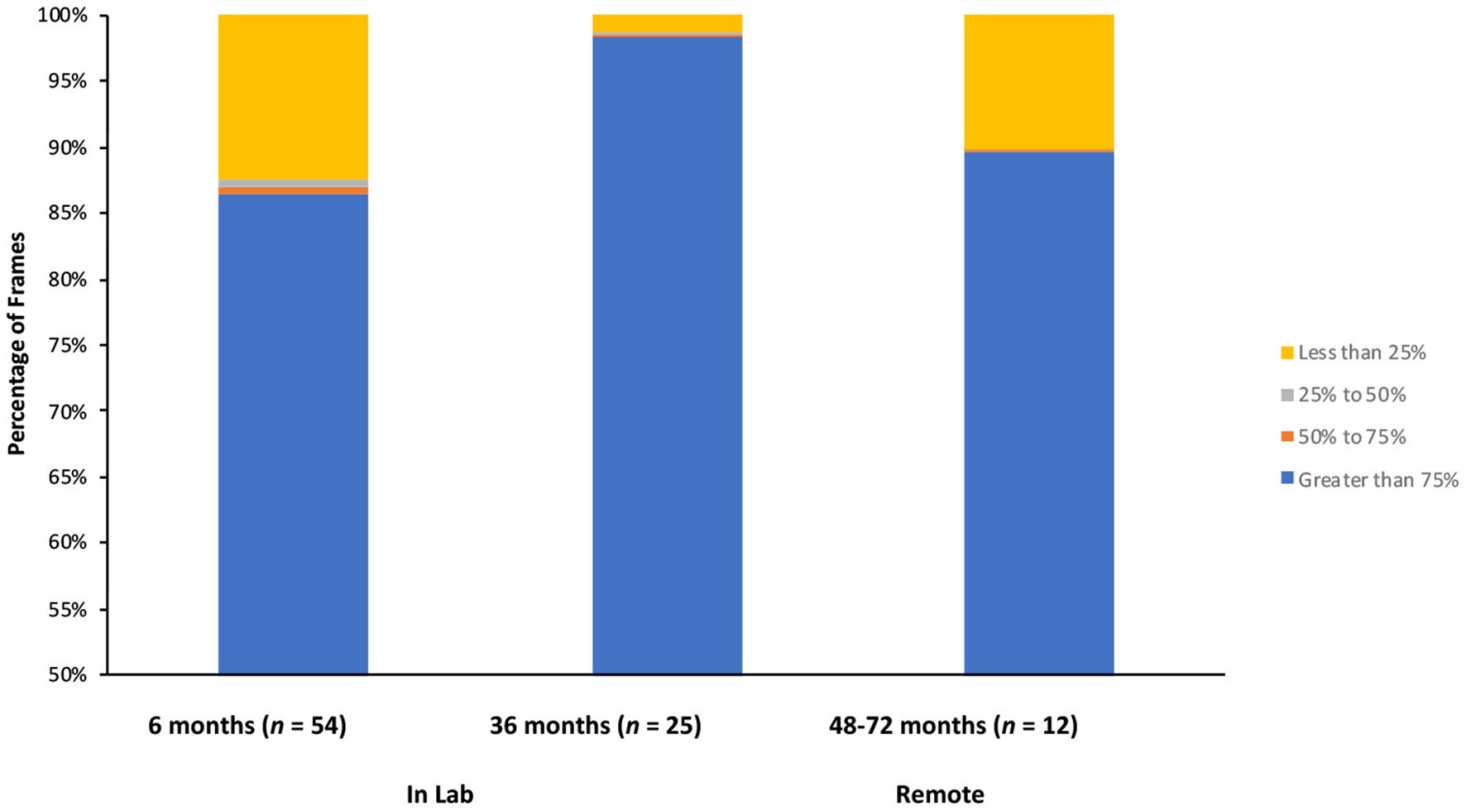
Distribution of confidence ratings: percentage of frames with confidence ratings of 75% or higher (in blue), 50–75% (orange), 25–50% (gray), or <25% (yellow) for 6-month-old infants (tested in the lab), 36-month-old children (tested in the lab), and 48–72-month-old children (tested remotely). For each participant, we calculated the percentage of frames in each quartile, and then averaged across participants to get these numbers.

Six- and 36-month-olds received the MAAP as a part of an ongoing longitudinal study. The longitudinal study, entitled “[blinded],” received IRB approval from the Social and Behavioral Review Board of [blinded] (IRB-13-0448-CR06). Video recordings of these sessions were processed by OpenFace as a proof of concept for this approach. Video data (including videos processed by OpenFace) are stored on a secure university server, can only be accessed by trained lab personnel, and can only be identified via a master key, which is kept in a separate, physical location. A summary of the confidence ratings can be found in Figure 2 and in general, were quite accurate. Averaged across all frames, 6-month-old infants (*n* = 54; tested in the lab) had an overall confidence rating of 83.68% (*SD* = 16.11%), 36-month-olds (*n* = 26; tested in the lab) had a confidence rating of 95.55% (*SD* = 3.37%). Further, inspection of the distribution of confidence ratings revealed that, at 6 months, 86.35% of frames analyzed by OpenFace had a confidence rating of 75% or higher, and at 36 months, 98.27% had a confidence rating of 75% or higher (see Figure 3).

### OpenFace Confidence Ratings for Data Collected Remotely

Video recordings of 48–72-month old children who participated in the MAAP remotely were also processed via OpenFace. Confidence ratings for children tested in the home (*n* = 12) were also quite accurate (see Figure 2 for a summary). Averaged across all frames, they had an overall confidence rating of 85.88% (*SD* = 18.51%). Further, inspection of the distribution of confidence ratings revealed that, 89.97% of frames analyzed by OpenFace had a confidence rating 75% or higher (see Figure 3).

### Challenges of Processing the Data

Because standard methods for coding looking time (see section Traditional Methods for Coding Looking Time for Infants and Children) are either too time consuming or impossible to use in a remote setting, we opted to automate the coding process, using OpenFace. OpenFace derives X, Y, Z (3D) coordinates of gaze direction and facial landmarks from the image of the participant's face on each video frame, but without translating these coordinates to an external frame of reference (i.e., locations on the participant's computer screen). This means that we don't know precisely where the participant is looking on the screen using the OpenFace output alone, given variability across participants in properties of the camera lens and visual angles. As a result, we developed a ML approach to overcome this lack of information about gaze direction with respect to the external frame of reference. To do this, we trained a ML model to classify gaze direction with respect to specific AOI on the screen, based on vector information provided by OpenFace. By training an ML algorithm in this manner, we can use the estimates provided by OpenFace to predict individual look directions and durations (traditional measures for looking time studies) for each participant (in our case, looking to the left, center, and right displays in the MAAP).

### Processing the Data Using Machine Learning and OpenFace

Machine learning is a data-driven approach for classifying patterns of relations between two or more variables typically from a large subset of a dataset (e.g., 50% of trials) in order to predict patterns of relations between these same variables in another subset of the data (e.g., remaining 50% of trials). ML algorithms accomplish this task by leveraging large amounts of data and computational power, and have been used in many disciplines (e.g., healthcare, autonomous driving, product recommendations). Artificial Neural Networks (ANNs) and their more advanced variants (Deep Neural Networks) are a widely used subset of ML approaches inspired by and based on biological neural networks. They are typically comprised of multiple connected node layers that translate a set of inputs (e.g., the X, Y, Z coordinates provided by OpenFace) into outputs (looks to left, center, and right displays on the MAAP). The “learning” or “training” process in ANNs is a powerful learning mechanism that can ultimately improve the accuracy of the network when presented with new data. This approach is similar to multi-voxel pattern analysis (MVPA) used to predict patterns of relations of fMRI and fNIRS data (e.g., Norman et al., 2006; Emberson et al., 2017). For example, MVPA can be used to predict neural activity in a single infant based on data patterns classified across the rest of the infants in a sample, or can predict patterns in one or more trials from patterns classified across the rest of the trials of a single infant (e.g., Emberson et al., 2017).

**Figure 4:**
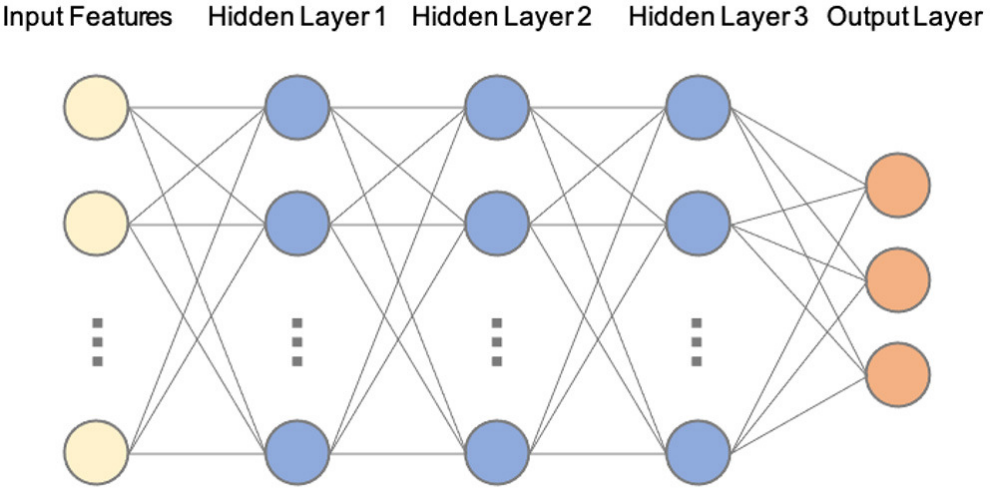
Example schematic of our current architecture of the artificial neural network (ANN). Our current ANN translates input features (from OpenFace) into three outputs (looking left, center, or right). Each node has an associated set of parameters (weights and biases) that generates an output. If the output of any individual node is above a specified threshold value, that node will pass data to the next layer of the network. Otherwise, no data will be sent to the next layer of the network. This powerful mechanism can be applied to modeling a plethora of problems that involve generating output values based on some input values. The “learning” or “training” process involves finding the optimum parameters for each node to minimize a cost function, which ultimately improves their accuracy. In order to improve the efficiency of model training, we included several standard regularization techniques. Specifically, each layer is followed by 1-D batch normalization (Ioffe and Szegedy, 2015) and a dropout procedure with the probability of 0.2 (Srivastava et al., 2014). We used the widely popular Rectified Linear Units (ReLUs) activation function (Nair and Hinton, 2010) at each node.

While there have been previous successful examples of using a combination of eye tracking and ML to estimate gaze localizations (e.g., George and Routray, 2016; Akinyelu and Blignaut, 2020; and for a review, see Klaib et al., 2021), our approach was developed specifically for our three-screen video based protocol to be used with infants and children with data collected remotely and thus complements these previous approaches. Our goal was to develop a ML model that could be easily used and understood by individuals with little or no prior ML modeling experience. As such, we chose to use a multi-layer ANN as our current ML algorithm (see Figure 4). After preliminary testing, we adopted a network consisting of four fully connected (three hidden) layers which was the minimum architecture effective for our specific needs. Our first model included one hidden layer. In subsequent model development, we tried a variety of layers and nodes (i.e., hyper parameters), ultimately settling on the three layers in the current model, which demonstrated excellent agreement with trained human coders. It should also be noted that we intentionally chose a simple architecture to evaluate the effectiveness of ML approaches in solving our unique problem. This simple yet effective neural network can also serve as a baseline for comparing the performance of more advanced deep learning techniques.

Here, we demonstrate the feasibility of using a simple ANN approach within individual infants, classifying patterns of relations between two variables (data coded by live observers and X, Y, Z coordinates provided by OpenFace) in a subset of the data (50% of video frames) in order to predict relations between these same variables in separate subset of the dataset (e.g., 50% of frames) from the same participant. Our goal is to design an algorithm that can use the information from a series of single images (one for each frame of our video recording) that is extracted when processing the video through OpenFace. Specifically, we use the following information from OpenFace as input to the model:

- Eye gaze direction vector and their average for both eyes
- Location of 2D and 3D eye region landmarks
- Pose estimates: location and rotation of the head with respect to camera
- Face Landmarks locations in 2D and 3D space
- Rigid and non-rigid shape parameters
- Facial Action Units.

More information about each one of the above inputs is available at https://github.com/TadasBaltrusaitis/OpenFace/wiki/Output-Format. The “learning” or “training” process in ML involves finding the optimum combination of parameters that can most efficiently predict an outcome (e.g., gaze direction).

## Objective 3: Training a ML Model to Calculate Looking Time Data

In order to calculate traditional looking time measures from gaze estimation vectors from OpenFace, we trained a ML model to classify gaze estimation vectors to looks to the left, right, and center displays of the screen (AOIs) during the MAAP protocol. We then compared ML estimates to estimates provided by human observers who coded data in the lab. For the data that were collected in the lab, look directions (to left, right, and center displays on MAAP trials) were coded live by trained observers according to standard procedures used in infant studies (e.g., Casey and Richards, 1988; Shaddy and Colombo, 2004; Bahrick et al., 2018a). Specifically, looking time and direction were coded by a primary and a secondary observer during task administration. Observers, hidden behind a black curtain, viewed the child through a front facing camera (SONY FDR-AX33) hidden above the widescreen monitor. Observers were blind to condition, and they coded infant fixations to the left, center, and right sides of the screen in real-time using a game pad. Button presses were fed into a custom computer program that calculated individual looking time to left center and right. Interobserver reliability was assessed by having the secondary observer record the looking for 66% of the participants (*n* = 36) at 6 months and 40% (*n* = 10) of the participants at 36 months. We assessed interobserver reliability by calculating the absolute difference between estimates of the two coders. To the extent that measures are free of random error (i.e., reliable), scores from each observer should be comparable (difference close to zero). This method is superior to correlational approaches for assessing inter-observer reliability, which are subject to artifacts (Goodwin and Leech, 2006; Jaccard and Becker, 2009). Inspection of the median absolute differences relative to the range of possible scores for each measure indicates little difference between the scores of the two observers and thus excellent reliabilities (differences were close to 0: from 0.009 to 0.053).

In order to train the model, we used looking time data that were coded live from the primary observer and then translated from individual look durations into frame-by-frame data. For this initial stage of ML training, we only used a subset of the total number of the 24 MAAP trials—a block of six social trials. All participants had a minimum of five out of six trials From these six trials, for each participant we randomly selected 50% of the 3,270 total frames for the ML training set and used the remaining 50% of frames for the testing set (to assess agreement between ML estimates and the estimates of the trained coder). For our next steps, we will use 50% of the entire 24 trials for the training set and the remaining 50% for the testing set to assess agreement. Our protocol was designed such that the size, location, and trial duration of left, center, and right displays are identical across social and non-social conditions. Thus, we anticipate strong agreement between ML estimates and a trained coder across all 24 trials (social and non-social) on the MAAP. Importantly, ML algorithms rely on multiple training iterations to learn and improve their accuracy, meaning the predictions should improve each time these training iterations are completed.

For the data that were collected online, because live coding was not possible, we assessed agreement between ML estimates of the child's looking behavior and the known locations of attention getting stimuli. We recorded videos of the participants watching attention getting stimuli and then used OpenFace to estimate gaze locations to the screen. The attention getting stimuli were presented in the middle of each of the AOIs (i.e., left, center, and right). They were presented one at a time, starting in the center, then, left, then back to center, and then to the right. This sequence was then repeated. Attention getting stimuli were presented for 1,500 ms each. Unlike previous attempts to localize gaze using ML (e.g., George and Routray, 2016), participants were not explicitly instructed to fixate each point. This was in part because our sample consisted of young children. Further, the attention getting stimuli were designed to be highly salient so that the children would fixate them. In addition to appearing rapidly, during the 1,500 ms presentation time, each point changed color, grew and then subsequently shrunk in size, and was accompanied by a series of salient sounds. This provided several known locations on the screen for each participant, for short periods of looking before the task started, serving as an external frame of reference. Importantly, when these attention getting stimuli were presented on the screen, they were the only thing visible. Therefore, we can assume that if the participant was looking at the screen, they were fixating each point. This allowed us to provide the model with a set of parameters for each of the three looking locations. Just like the approach described above for the data collected in the lab, 50% of data for each participant was used for training and validation of the ML algorithm and the rest was used for testing the performance of the algorithm.

After demonstrating the effectiveness of using OpenFace to quantify looking time in infants and young children by using the face, head position, and eye gaze direction of the participants (section OpenFace Confidence Ratings for Data Collected In-lab), we then compared the gaze estimate (provided by OpenFace) to a known location. To evaluate these outputs, we computed a percent agreement rate, or the number of frames where the OpenFace output provided the same estimate (e.g., left, center, right) as the live coder or as the known location (attention getting stimuli), divided by the total frames. For the data that were collected in the lab, we used data that were coded live (during data collection), and for the data collected remotely, this consisted of using attention getting stimuli (to provide a ground truth).

### Predicting Data Collected In-lab

**Figure 5:**
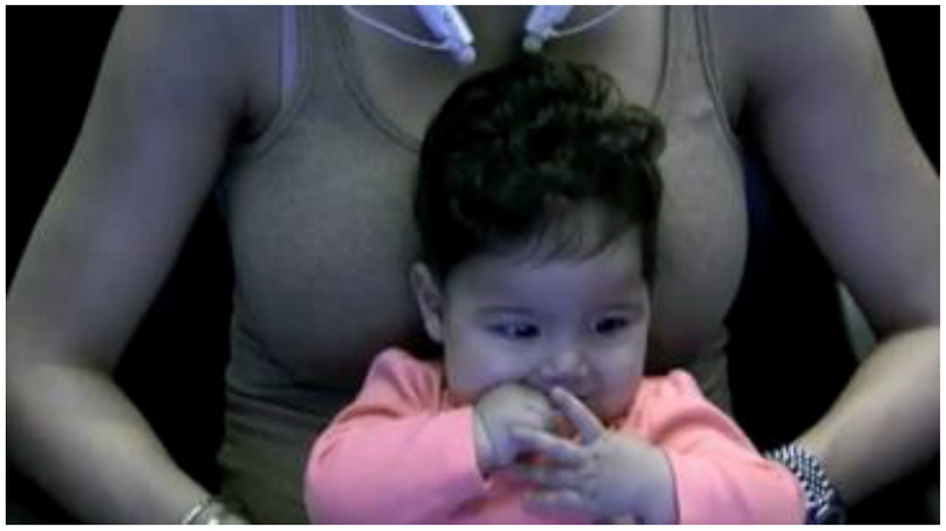
Participant A (in-lab). Example of a 6-month-old infant that OpenFace may have trouble identifying all components (facial landmark, head pose estimation, facial action unit recognition, and eye-gaze estimation) due to obstruction (hand) of the child's face.

**Figure 6:**
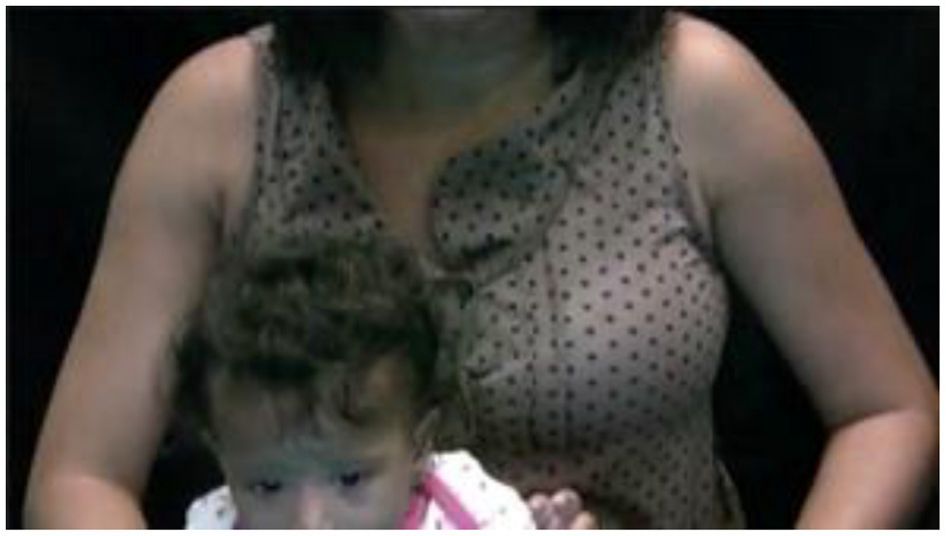
Participant B (in-lab). Example of a 6-month-old infant that OpenFace may have trouble identifying all components due to the fact that the video did not capture all of the child's face.

**Figure 7:**
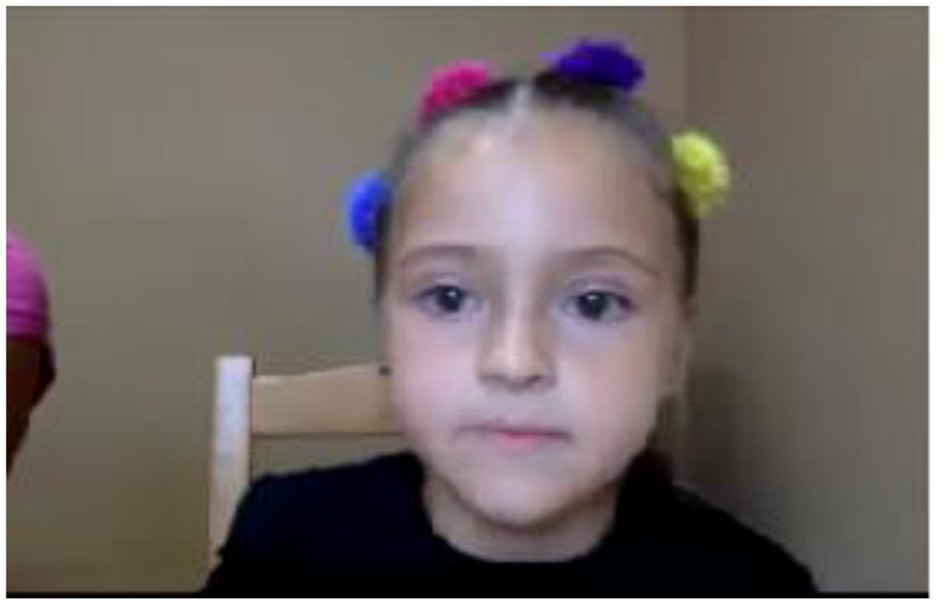
Exemplary video for OpenFace.

For data collected in the lab, agreement between predictions of the ML model and live coders was calculated on the 50% of frames not used for training (i.e., the testing set). Assuming an equal distribution of looks to left, center, and right, without model training, the initial predictions of the model should be at chance (33%). However, training the model should result in dramatic improvement in agreement. For example, following model training, for 6-month-old infants (*n* = 54), the ML model had an average agreement rate of 89.9% with the live coders (*SD* = 6.75%). Further, this result was not driven by any one location on the screen as agreement scores were 82, 88, and 87% to the left, center, right, respectively. There were no significant differences in agreement among left, center, and right displays (*p*s > 0.199). It is important to note that because the amount of looking to center, right and left locations differed, the average agreement rate computed was a weighted average and thus does not correspond precisely to the mean derived by averaging across the scores for the three locations (left, right, and center displays). Similarly, for 36-month-olds (*n* = 25), the ML model had an average agreement rate of 85.83% with the live coders (*SD* = 5.85), with 85, 86, 80% agreement to the left, center, and right (with marginally greater agreement to the center display, 86%, than right display, 80%, *p* = 0.092). Two 6-month-old infants had very poor agreement to individual locations. Participant A had only 31% agreement to the center location and Participant B had only 0.05% agreement to the left location. Further inspection revealed that OpenFace appeared to have difficulties identifying all of the necessary input components due to the fact that Participant A's hand was obscuring the face for large parts of the task (see Figure 5) and Participant B's face was not entirely in the video for large sections (see Figure 6). An optimal set up can been seen in Figure 7. With these two individuals removed from the dataset, average percent agreement for the 6-month-old infants improved to 90.18% (*SD* = 6.63%; Table 1) and looking the left, center, and right improves to 84, 89, and 87% agreement, respectively. Therefore, in developing inclusion criteria for this and future attempts using OpenFace, one should ensure that participant faces are fully visible.

**Table 1:** In lab ML agreement without participants A and B.

**Age (months)**	***n***	***M* (%)**	***SD* (%)**	**Range (%)**
6	52	90.18	6.63	73.51–99%
36	25	85.83	5.85	72.57–95.12%
Total	77	88.77	6.67	72.51–99%

### Predicting Data Collected Remotely

Because the data collected remotely could not be coded live, the ML model used attention getting stimuli (at the beginning of each block) as a frame of reference, similar to the procedure used by most remote eye-trackers.

**Figure 8:**
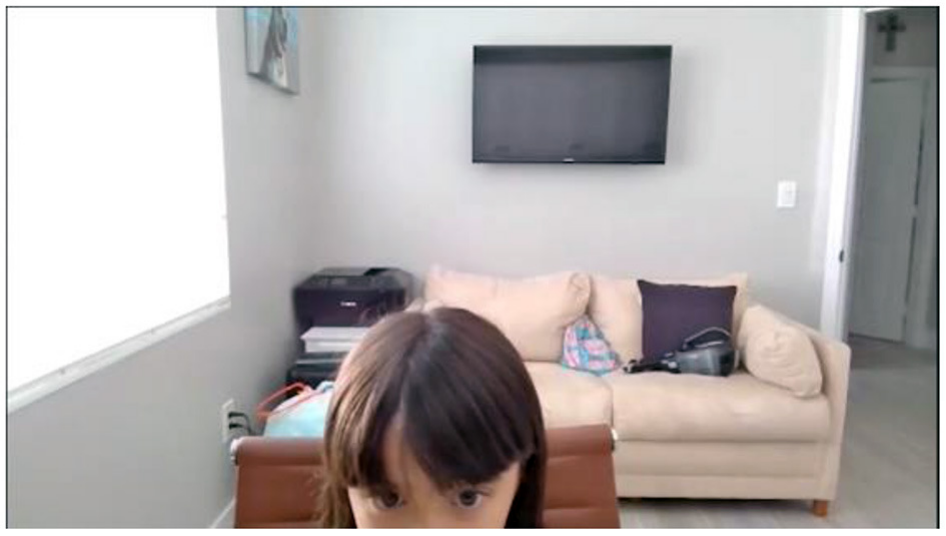
Participant C (at-home). Example of a 72-month-old child that OpenFace may have trouble identifying all components due to the fact that the video did not capture all of the child's face.

Following training, the average percent agreement for data collected remotely (48-, 60-, and 72-month-old children; *n* = 12) between the ML model and the attention getting stimuli was 85.63% (*SD* = 24.76%) with 82, 84, and 83% agreement to the left, center, and right locations. There were no significant differences in agreement among left, center, and right displays (*p*s > 0.552). One participant had an average agreement score of 7.85%. Again, this appeared to be due to the fact that the child's face was not entirely in the frame while the attention getting stimuli were being presented (see Figure 8). When that participant was removed (Participant C), average agreement between the ML model and the attention getting stimuli improved to 90.7% (*SD* = 7.06%: Table 2) and to 89, 91, and 88% for looking to the left, center, and right. Therefore, when the full face is within view during calibration, the ML model seems to have excellent potential for translating the OpenFace output into gaze locations to specific AOIs on the screen for individual participants. Thus, again when using OpenFace, an important inclusion criterion should be that participant faces are fully visible.

**Table 2:** Online ML agreement without participant C.

**Age (months)**	***n***	***M* (%)**	***SD* (%)**	**Range (%)**
48	2	89.73	8.87	83.45–96
60	3	95.14	2.68	92.07–97.06
72	6	88.8	8.03	76.43–96
Total	11	90.7	7.06	76.43–97.06

### Demographic Differences

Because facial recognition software can sometimes be biased toward or perform better with members from the ethnic or racial group that developed it (e.g., Mehrabi et al., 2019), we explored the ability of OpenFace to identify and predict gaze across individuals who differed in gender, ethnicity, and race. Importantly, the ability of OpenFace to identify facial landmarks (i.e., confidence ratings) did not significantly differ as a function of gender (*p* = 0.761), Race (*p* = 0.227), or Ethnicity (*p* = 0.170). Additionally, percent agreement between the ML model and estimates from in-lab and remotely administered experiments did not significantly differ as a function of gender (*p* = 0.219), Race (*p* = 0.189), or Ethnicity (*p* = 0.520). See Tables 3–5 for means and standard deviations.

**Table 3:** Confidence rating (OpenFace) and ML agreement as a function of gender.

	**Gender**	***N***	***M* (%)**	***SD* (%)**
Confidence rating	Male	45	87	15.90
	Female	37	86	15.45
ML agreement	Male	45	89	6.94
	Female	37	86	14.65

*OpenFace provides a confidence rating for every frame of a video (using all face, head position, and gaze direction landmarks). The confidence rating is a measure of how well OpenFace can identify all of these components (averaged across all frames for each participant)*.

**Table 4:** Confidence rating (OpenFace) and ML agreement as a function of race.

	**Race**	***N***	***M* (%)**	***SD* (%)**
Confidence rating	African American	11	77	24.69
	White	57	87	14.33
	Other	2	80	8.88
	More than 1 race	6	91	11.04
	DNA	6	94	3.11
ML agreement	African American	11	93	4.21
	White	57	85	12.46
	Other	2	87	8.71
	More than 1 race	6	91	5.74
	DNA	6	93	11.13

**Table 5:** Confidence rating (OpenFace) and ML agreement as a function of ethnicity.

	**Ethnicity**	***N***	***M* (%)**	***SD* (%)**
Confidence rating	Hispanic or Latino	53	89	12.01
	Not hispanic or Latino	28	81	20.66
	DNA	2	88	13.58
ML agreement	Hispanic or Latino	53	87	13.04
	Not hispanic or Latino	28	90	6.66
	DNA	2	88	2.43

## Discussion

In this article, we have described our novel and successful method of data collection during the COVID-19 pandemic. While there are inherent challenges to testing remotely, and even more challenges when testing children, we found that with the proper attention to detail, very good quality data can be collected. Using a combination of Zoom and Gorilla Experiment Builder, looking time tasks can be programmed and used in the home easily and efficiently. Importantly, Gorilla afforded us with the level of audio-visual precision that was necessary for our looking time task. We also found it important to provide the caregiver with explicit instructions during a pre-session for how to help with data collection and serve as the “at-home experimenter” and facilitate testing without interfering. Once the data were collected, we used OpenFace (an open source gaze estimation tool) and a ML model to process the data. We developed a ML approach that was intentionally simple (compared to other deep learning techniques). We first tested this approach using data that were previously collected (and coded live by human observers) in the lab and then applied the approach to data collected remotely.

Our results revealed that the overall agreement between the live observers and the ML model was high (~90% for 6- and 36-month-olds) suggesting that the combination of OpenFace and ML performs at a level similar to well-established methods for collecting looking time data. After demonstrating that OpenFace could be used to estimate gaze for infants and children in a lab setting, we expanded our dataset to include children tested remotely in the home. Because these data could not be coded live by observers in real time, we used attention getting stimuli to compare the ML model's predictions of gaze locations to known locations. Once again, the ML model's estimates of gaze locations had high agreement (~90% for 48-, 60-, and 72-month-olds) with that of the known locations on the screen, demonstrating that this method is suitable for estimating gaze direction for data collected remotely.

## Future Directions and Limitations

While we have demonstrated initial success in implementing this novel approach, it is important to note that both data collection and model development are ongoing. Further, we should acknowledge, that while results of our ML model are promising, we are just beginning to test its effectiveness with infants. Preliminary results look promising. Thus far, we have tested four infants online (*n* = 2 at 15 months, *n* = 1 at 13 months, and *n* = 1 at 4 months). We have processed their video data in OpenFace. Mean confidence ratings were as follows: 15-month confidence ratings = 90.39%, 87.46%, 13-month confidence rating = 70.63%, 4-month confidence rating = 68.73%. While the 15-month data look strikingly similar to the average of our online data, the confidence ratings for the 13- and 4-month-old infants were slightly lower. Again, we should also note, there were no initial criteria in place for data quality of the videos processed in OpenFace. As such, we are confident that with further development, once factors such as looking away from the screen or the face being obscured are taken into account, confident ratings will increase. Future research should take this into account.

One current limitation of our approach is that the ML model requires training on a subset of the data. However, our ultimate goal is to establish a fully autonomous model that is robust enough to classify gaze without training on a subset of the data. As our data set continues to grow, so too will the generalizability of our ML model. In addition, we plan to train more complex models with a larger number of parameters once we incorporate additional data from multiple participants. This involves adding more layers and more neurons to our neural network. Specifically, we will use K-fold cross validation for training the model. This works by randomly dividing the training data set into groups (folds) and repeating training steps K times (where K = the number of iterations) while at each time we hold out one of the sections of data (folds). This allows us to test the accuracy of the model on multiple sections of the dataset, increasing our overall accuracy estimates.

Another limitation of our current approach is that it was developed specifically for coding data from a three-screen audio-visual protocol (the MAAP). Once we incorporate all the data from multiple participants for training, the ML model will be able to be used for various types of input (i.e., other looking time tasks), and can be scaled up to incorporate more complex gaze estimates (e.g., more than three locations). Our lab is currently adapting a ML model to be used with the Intersensory Processing Efficiency Protocol (IPEP; Bahrick et al., 2018b), an audiovisual task similar to the MAAP, but with six AOIs as opposed three.

Recently, the approach that has been outlined in this manuscript has been adapted by the Multisensory Data Network (a collection of 13 research labs across North America) who will administer the MAAP and IPEP remotely, process the looking time data using OpenFace and our ML model, and add to our growing dataset, helping to further inform and refine our model as well as develop preliminary norms for the development of skills assessed by the MAAP and IPEP. We plan on publishing our dataset (once it is complete) for scientists to use. As such, this paper provides an important first step in developing an open source toolkit capable of quantifying large-scale looking time data collected remotely for infants and children.

Finally, we acknowledge that the minimum technological requirements of a home computer with a web camera and high-speed internet connection could potentially limit our participant pool. Specifically, as Lourenco and Tasimi (2020) have recently pointed out, the technological requirements of many online studies may restrict access to many low-income and minority communities and thus, may impact the generalizability of the findings.

## Conclusions

These preliminary results suggest that under the right circumstances, OpenFace can be used with infants (both with data collected in a lab setting and preliminary data collected remotely) and with young children (for tasks that were administered remotely) to derive gaze vectors for looking time. Further, when paired with our ML model, we can accurately and efficiently process looking time data and provide an output that is comparable in accuracy to traditional methods of looking time. As such, our approach provides developmental researchers with a viable option for collecting looking time data outside of the typical laboratory setting. Not only does this provide researchers with a cost-effective method for data collection, but it also frees them from the geographical confines of testing individuals within the typical university community, opening the door to a world-wide participant pool.

## Data Availability Statement

The raw data supporting the conclusions of this article will be made available by the authors, without undue reservation.

## Ethics Statement

The studies involving human participants were reviewed and approved by Florida International University Office of Research Integrity. Written informed consent to participate in this study was provided by the participants' legal guardian/next of kin. Written informed consent was obtained from the individuals and minor(s)' legal guardian/next of kin for the publication of any potentially identifiable images or data included in this article.

## Author Contributions

BE, JT, and LB developed the study concept. BE, JT, AS, and LB contributed to the study design. EE, VP, MM, and WG performed data collection and coding. BE, JT, and AS performed the data analysis and interpretation. BE drafted the manuscript, JT, AS, and LB provided critical revisions. All authors contributed to and approved the final version of the manuscript for submission.

## Funding

This research was supported in part by the National Institute on Minority Health and Health Disparities of the National Institutes of Health Under Award Number NIMHD (U54MD012393), Florida International University Research Center in Minority Institutions, awarded to BE. This work was also supported by the NICHD: Grants R01-HD053776 and R01-HD094803 awarded to LB.

## Author Disclaimer

The content is solely the responsibility of the authors and does not represent the official views of the National Institutes of Health.

## Conflict of Interest

The authors declare that the research was conducted in the absence of any commercial or financial relationships that could be construed as a potential conflict of interest.

## Publisher's Note

All claims expressed in this article are solely those of the authors and do not necessarily represent those of their affiliated organizations, or those of the publisher, the editors and the reviewers. Any product that may be evaluated in this article, or claim that may be made by its manufacturer, is not guaranteed or endorsed by the publisher.

## Footnotes

^1^Edgar, E. V., Todd, J. T., and Bahrick, L. E. (Under review). *Intersensory Matching of Faces and Voices in Infancy Predicts Language Outcomes in Young Children*. [Manuscript submitted for publication]. Department of Psychology, Florida International University.

^2^Todd, J. T., and Bahrick, L. E. (Under review). *Individual Differences in Multisensory Attention Skills in Children with Autism Spectrum Disorder Predict Language and Symptom Severity: Evidence from the Multisensory Attention Assessment Protocol (MAAP)* [Manuscript submitted for publication]. Department of Psychology, Florida International University.
