# Supplementary material for: Remote Data Collection During a Pandemic: A New Approach for Assessing and Coding Multisensory Attention Skills in Infants and Young Children
Source: Front Psychol. 2022 Jan 21;12:731618. doi: 10.3389/fpsyg.2021.731618 (PMC8815727; doi:10.3389/fpsyg.2021.731618)
Supplement: Supplementary file 1 [file Presentation_1.pdf]

1 Supplementary Material

1.1 Programming the MAAP in Gorilla

Because the MAAP requires precise temporal alignment of the audio and the video, it was important to achieve the precision of the lab, but in an online format. In the lab, specialized computer software can time-lock trial onsets and off-sets with observer button presses. Unfortunately, this approach caused some timing issues and lagging when constructed in Gorilla, causing some trials to lose their audio-visual synchrony. We found that stitching all of the 24 trials of the MAAP into a single .mp4 file provided us with consistent presentation of the task (e.g. persevered the audio-visual synchrony), even when presented remotely. This allowed us to minimize any potential delays due to network speed, as the resources from the entire task were loaded in advance of presentation, a process called caching. While our current approach makes designating trial onsets/offsets slightly more difficult, we are able to identify trials onsets/offsets with relative ease, using Adobe Premier Pro's "synchronize on audio" feature. Specifically, using the individual MAAP videos (unstitched) as a reference, we use Adobe Premier Pro's "synchronize on audio" feature to identify where each trial begins and ends. In the event Adobe is unable to synchronize (i.e. baby cooing, crying, speaking over the audio track) due to a disruption during the recording we can manually synchronize using the audio waveform (a visual representation of the recorded audio), and confirm synchrony by listening to the audio playback until all trials are synchronized. In addition, we were able to compress the video files, reducing the strain on the participant's computer processor. This reduced the total loading time and ensures consistency across multiple systems and varying internet speeds. Finally, because we standardized the size of the videos in Gorilla, the only requirement for screen size was that it be at least 11 inches.

1.2 Zoom settings necessary for consistent audio-visual presentation

In order to maintain the level of precision needed for this task there are a number of settings that the experimenter needs to enable. The first is the ability to "record active speaker, gallery view and shared screen separately". This feature allowed us to record a high-quality video of the participant in a sufficiently large size so we could code eye gaze. Without this setting, the video of the participant is very small and of low quality, making data coding difficult. The second setting that is necessary for high-quality data collection is the ability to "record to the cloud". In addition to providing a safe place to store video data, this setting allowed us to quickly and efficiently download high quality videos that maintained audio-visual synchrony. It is also necessary to "optimize for third party video editor" when recording the session. This ensures data quality and optimizes the timing of the participant's behavior, ensuring that it aligns with the timing of the audio-visual display. synchrony of the participant's behavior and the video displays to allow us to code accurately. For example, reaction time to look to an event might be distorted if this were not enabled. Note: Zoom updates their software frequently and at the time of publication, we were referencing Zoom version 5.6.4 (765).

1.3 Technical requirements

1.3.1 Experimenter

- Computer with webcam
- Two experimenters:

    - It is best to have two experimenters conduct each session
    - A second experimenter can serve as a backup if the primary experimenter has internet connectivity issues and is needed to enable the spotlight function on Zoom to ensure the participant's face stays in view the entire session
- Stable internet connection (download: > 100 Mbps is good; ping: < 20 ms)
- Latest version of Zoom account. For information on how to check for updates: https://support.zoom.us/hc/en-us/articles/201362233-Upgrade-update-to-the-latest-version

1.3.2 Participant

- **Computer with a webcam**
    - Desktop or laptop with a centered webcam located at the top of the screen (tablets are problematic and not recommended)
    - We do have the ability to "enhance" certain videos if webcam quality is bad
- **Internet Connectivity/Speed:**
    - Download: Greater than 100 Mbps is excellent, between 50-100 Mbps is good. Anything below 50 Mbps may cause issues, but you should still try to run the session.
    - Ping: Less than 20 ms is good. Greater than 25 ms may cause issues.
- **Operating system**: Gorilla, Q-global, and REDCap are compatible with Mac and Windows OS
- **Screen size**
    - Need at least an 11" screen, but bigger is fine
    - To standardize the visual angle, the MAAP and IPEP videos will appear at a constant size regardless of screen size
    - Note: Participants must sit at a standard distance from the screen, 12-15 inches
- **Browser:**
    - Mac: Chrome is preferable, but Safari and Firefox will also work
    - PC: Chrome is preferable, but Firefox will also work
    - Not advised: Internet Explorer or Edge
- **Latest Version of Zoom:**
    - Parents should have the most recent version of the Zoom application
    - For information on how to check for updates: https://support.zoom.us/hc/en-us/articles/201362233-Upgrade-update-to-the-latest-version
- **Tape measure**: Parents should have tape measure or ruler available on the day(s) of testing to measure the distance between the child and the screen

1.4 Instructions to parents: Pre-session

1. Ask the parent to close any web browser windows and/or computer programs they might have open.

"Can you close out of all other computer programs and web browser tabs and windows before we begin?"

2. Ask the parent to have a tape measure nearby and ensure that the child is 12 to 15 inches from the screen (see section Pre-Session -> Camera Angles, for examples):

"Do you have a tape measure handy? If not, now is a good time to go get one. We have to ensure that your child's face is close enough to the screen so that we get a good, high-quality video."

3. Check to see if video is mirrored: Ask the parent to lift their right hand and then their left hand. Make a note on the MAAP/IPEP cover sheet (e.g., Parent left is Experimenter right).

"Can you please lift up your right hand? And then your left hand? Thanks!"

4. Before sending the Gorilla MAAP/IPEP link, tell the parent:

"Now your child will watch some videos of women talking and objects hitting a surface. The videos will last about 8-10 minutes. During the videos, we ask that you try to not distract your child or direct their attention to the screen. We are interested in how children naturally attend to these events without instructions. If we need your child to move closer to the screen, we will let you know."

5. Ask if the child will sit by themselves or in the parent's lap:

If the child is sitting in the parent's lap:

"We have two options you can choose from to prevent you from seeing the videos. First you can create a pair of blinder glasses by covering the lenses of a pair of glasses or sunglasses with either sticky notes or small pieces of paper taped to the lenses. The second option, which allows you a better view of your child, is to create a paper shield. You can do this by simply securing a sheet of paper under the rim of a hat (backwards baseball cap works well) or headband so that it covers your face but allows you to look downward. Which do you prefer?"

If the child is sitting by themselves:

"During the test session, we would like for you to site outside of your child's view, either to the side or behind them, and not watch the videos. We also ask that you not gesture or speak to the child during the tasks as we need your child to answer everything on their own. Finally, we ask that you please avoid phone calls, texting, or other disruptions so that you can play an active role as our At-Home Experimenter."

6. Copy and paste the Gorilla link in the Zoom chat and hit send

"Do you see a link in the Zoom chat? Great! Please copy and paste this link into an empty browser window. Please remember to paste this link into either Chrome, Firefox, or Safari."

After the web page has fully loaded:
"Please maximize the window of your web browser so that it takes up your full screen. I want you to leave it this way the entire time."

7. Once the parents have opened the Gorilla link, ask them to share screen with Zoom:

"Before you fill out the questions that appear on your screen, I need you to first share your screen and computer audio with me."

"First, go back to Zoom and look for the green "Share Screen" button at the bottom of the window."

Remote data collection

“Once the window has popped up, look for two options located on the bottom left of the window. I want you to select “Share sound” and “Optimize for video clip”

“Now select “Screen” and click the blue “Share” button on the lower right side of the window.”
